# Supplementary material for: Spatialtemporal evolution characteristics of ozone in China and its response to urbanization
Source: PLoS One. 2024 May 31;19(5):e0300185. doi: 10.1371/journal.pone.0300185 (PMC11142485; doi:10.1371/journal.pone.0300185)
Supplement: Attachment — Submitted filename: Response to reviewers .doc [file pone.0300185.s003.doc]

Thank you for your letter and for the reviewers' and editors' comments concerning our manuscript. These comments are very helpful for revising and improving our paper. We have studied comments carefully and have made corrections which we hope meet with approval.

**Reviewer #1comments1:**

The introduction needs to be improved, and it is necessary to expose the innovations that this study brings compared with other urbanization or air pollution studies. We also encourage the authors to add some references to improve the introduction according to the research gaps and the needs of the study.

[1] Cao N, Luo N, Peng Y, et al. Evaluation of Urbanization Development Dynamics Based on Quadrilateral Diamond Structure Model: Evidence from Chongqing, China, in the New Era[J]. Journal of urban planning and development, 2023,149(3). <https://doi.org/10.1061/JUPDDM.UPENG-4413>

[2] Sun P, Luo N. Comparative Analysis on the Quality of Urbanization Structure of Central Cities in Southwest Economic Core Area of China: Taking Chengdu-Chongqing as Examples[J]. Scientia Geographica Sinica, 2021,41(6): 1029-1039. DOI:10.13249/j.cnki.sgs.2021.06.011

**Response:** Thanks for your valuable comments and suggestions. We have improved the introduction by adding innovations brought by this study compared to urbanization or air pollution studies. We have improved the introduction by adding innovations brought by this study compared to urbanization or air pollution studies. At the same time, the following references were added to supplement the introduction based on research gaps and research needs. The details are as follows:

Urbanization is the process of economic, social, land, and population transformation from countryside to towns in the course of a country's development. Among them, the core of urbanization is population urbanization, and the carrier of urbanization is land urbanization [11]. With the intensification of atmospheric environmental pollution, scholars from all walks of life have studied the effect of pollution reduction and carbon reduction from multiple perspectives and found that urbanization is an important factor affecting the atmospheric environment [12]. "China's New Urbanization Report 2012" points out that the non-intensive and crude production of China's urbanization focuses on the quantity and scale of urban development, which ignores the value of environmental resources and is an important cause of the decline in the quality of the atmospheric environment. The problem of air pollution in China has changed from single pollutant pollution to compound pollution, which is increasing with the rapid development of urbanization in China [13] [14]. Civerolo et al. [15] used the SLEUTH model to extrapolate New York City's land cover data from 1990 to the next 2050 and predicted that future urbanization in New York will lead to elevated ozone concentrations. Scholars agree with the conclusion that urbanization has an impact on air pollution. Carbon emissions and haze are the main control gases. Scholars from all walks of life have done a lot of research on them, and the methods are diverse. From the existing literature, the STIRPAT model [16], Dubin model [17], DPSIR model [18], the geographically weighted regression model (GWR) [19], geographical detector [20], and so on are mainly used to study the development law of air pollution. Chameides [21] believed that the urban heat island effect caused by urbanization is the main reason for the increase in urban ozone concentration. Many scholars use the WRF Chem model to check ozone pollution, and it shows that urbanization increases ozone concentration. [22-23]. Overall, the existing research results are relatively rich and provide an empirical basis for exploring the relationship between urbanization and air pollution. However, the exploration of urbanization on ozone pollution is still relatively small. In recent years, the causes of atmospheric pollution have strengthened from a purely environmental problem to a complex ecosystem problem of population, land, economy, and society. Current urbanization studies mostly use land cover data to select indicators and fail to consider other factors that influence ozone concentration. There is also limited research on the atmospheric environment effect from the perspective of urbanization. Therefore, understanding the characteristics of spatial and temporal changes in ozone and the driving mechanisms from the perspective of urbanization is essential to further promote the harmonious development of nature and society.

Considering all the above explanations, the present study proposes to address the following questions: i) The distribution characteristics of ozone in time and space. ii) Under the background of urbanization development, the driving mechanism of ozone pollution is analyzed (from the four urbanization development directions of economy, population, land, and society). iii) To unify the distribution of factors affecting ozone concentration by urbanization development in all provinces of the country. In addition, the distribution and driving mechanisms of ozone pollution from the perspective of China's urbanization are less frequently studied. To answer these questions above, we attempt to use ozone concentration data and statistical yearbook data of 31 provinces in China to study the driving mechanism of ozone pollution under the context of urbanization. We intensify on the impact of urbanization indicators on ozone and analyze the geographical spatial distribution of urbanization impact factors on the ozone impact index. By citing urbanization, hope to be more comprehensive on the causes of China's distribution characteristics of ozone pollution and how to solve this problem are discussed, and thus draw more accurate results and set forward more constructive suggestions. The intensification of atmospheric environmental pollution in China has attracted extensive attention from the government and academia, but the domestic existing literature, focuses on exploring the micro-channels of urbanization on ozone pollution, and the index selection is based on urban land cover data. The research on the impact of other influencing factors of urbanization on ozone concentration is indeed more insufficient. Therefore, this study aims to empirically analyze the impact mechanism and driving factors of ozone pollution in the context of urbanization in China using the dynamic spatial Durbin model and the spatio-temporal geographically weighted regression model. Producing a new explanation for the causes of the spatial and temporal distribution characteristics of ozone pollution, producing a basis for regional ozone and pollution prevention and control measures, has important theoretical and practical relevance for the government to formulate and improve atmospheric environmental protection policies, and how the government will carry out macro in the future.

Thanks!

**Reviewer #1comments2:**

Please ensure the accuracy of data sources: the author uses air pollution data at the prefecture-level city, so the process of aggregating such data to the provincial level requires further explanation. Also, the URL in line 197 contains errors, please check and correct it.

**Response:** Thanks for your valuable comments and suggestions. We rewrote the data source and processing to correct the URL in line 197. The details are as follows:

**Data Sources**

Based on the inter-provincial panel data from 2015 to 2019, this paper takes 31 provinces and regions in China as research samples. According to the historical data of 268 prefecture-level cities released by China Air Quality Online Monitoring and Analysis Platform (<https://www.aqistudy.cn/historydata/>), the concentration data of O_3_, NO_2_, CO, SO_2_, PM_10_, PM_2.5_ and AQI in each prefecture-level city were integrated at the provincial level, and the annual average value was used to calculate the concentration data of air pollutants in each province. O3 (8h) is the ozone concentration data obtained by 8-hour ozone moving average concentration detection. We derived the data of urbanization variables and control variables from the National Bureau of Statistics (<https://data.stats.gov.cn/easyquery.htm?cn=C01>), and we derived all other relevant data from the statistical yearbooks of Chinese provinces.

Thanks!

**Reviewer #1comments3:**

"Fig 1. Flow chart of urbanization ozone pollution" needs correction. The relationships and connections between different components in Fig. 1 are not clearly characterized. I suggest the author further refine the contents in this figure, especially by establishing clearer links between aspects of urbanization and specific variables (e.g. industrial structure, urbanization rate). This could better explain the rationale behind selecting the independent variables, and illustrate the mechanism linking urbanization and ozone pollution.

**Response:** Thanks for your valuable comments and suggestions. The specific variable indicators included in the demographic urbanization, land urbanization, social urbanization, economic urbanization and control variables are indicated by using the form of arrows and the same shape of the outer frame of the variable. The details are as follows:

**Fig 1. Flow chart of urbanization ozone pollution**

Thanks!

**Reviewer #1comments4:**

The "Characterization of ozone spatio-temporal evolution" in the Empirical Results section does not combine time and space in the analysis, making the contents and results less clear. I suggest the author reorganize this part, for example, combining Table 3 and Fig. 3 to present the results on a map, which could better demonstrate the characteristics of different clustering types.

**Response:** Thanks for your valuable comments and suggestions. We redrew a map of the spatial distribution of ozone (Figure 3), which can better demonstrate the characteristics of different clustering types. And we use a combination of time and space for the analysis. The details are as follows:

The results of Figure 3 further show that the distribution characteristics of near-surface ozone in China are "east-heavy, west-light," "short in the south and high in the north". The spatial distribution trend of diffusion to central China and coastal cities is more prominent in the Beijing, Tianjin, Hebei, and Shandong provinces. Significant spatial heterogeneity characterizes the inter-province. Hebei Province, Tianjin City, and Shandong Province are the key areas of the petrochemical and organic chemical industry in China, and the stock of coal-fired boilers is relatively large. The region has dense traffic, developed logistics, and huge emissions of diesel trucks and fuel vehicles. These factors have expanded VOCs and nitrogen oxide emissions in these areas, forming a medium and long-term pressure on ozone prevention and control in the region. In 2015, the high-value areas of ozone concentration in China were distributed across the eastern region, mainly in Beijing, Shanghai, Shandong, Jiangsu, Qinghai, Zhejiang, Gansu, and Inner Mongolia. In Shanghai, the average annual concentration reached 105*μg/m^3^*. By 2019, the concentration of ozone in China increased compared with 2015, and ozone pollution expanded rapidly in the east, mainly concentrated in North China and East China. Among them, Hebei Province, Tianjin City, and Shanxi Province in North China are the high-value areas of ozone pollution concentration growth in China. The near-surface ozone concentration in Tianjin increased from 77 *μg/m^3^* in 2015 to 106 *μg/m^3^* in 2019. This is because more developed industries, dense populations, and higher emissions of ozone precursors from motor vehicles and industrial sources typically characterize these areas [5].

**Fig 3. The spatial distribution of ozone concentration in China's provinces in 2015 and 2019**

Thanks!

**Reviewer #1comments5:**

In the Response Results section, please add validation results of the spatial econometric models (in a table form); also, only very few indicators in Table 4 pass the significance test, and the Coefficients and t-values are inconsistent in signs, please double check and correct these issues.

**Response:** Thanks for your valuable comments and suggestions. We have added a table of LM test results to the Response Results section and corrected the contents of Table 5. The details are as follows:

**Response Results**

In order to determine the specific form of the model, this paper carried out the LM test, LR test, and Hausman test. According to the LM results in Table 3, LM rejects the null hypothesis, and the results tend to be the spatial error model (SEM). Further, the LR test is used to verify the model, and it is found that the null hypothesis is rejected at the 1% level. The Hausman test showed that the null hypothesis was rejected at the 1% level. Therefore, the fixed effect spatial error model (SEM) was finally selected for the experiment.

**Table 3. LM test of spatial panel model**

| LM test | | | |
| --- | --- | --- | --- |
|  | Statistic | df | p-value |
| Spatial error |  |  |  |
| Moran’s *I* | 113.175 | 1 | 0.000 |
| Lagrange multiplier | 8.510 | 1 | 0.004 |
| Robuet Lagrange multiplier | 8.680 | 1 | 0.003 |
| Spatial lag |  |  |  |
| Lagrange multiplier | 0.826 | 1 | 0.364 |
| Robuet Lagrange multiplier | 0.995 | 1 | 0.319 |

**Table 4.** Spatial error model under nested matrix

| Variable | Direct effect | | Indirect effect | | Gross effect | |
| --- | --- | --- | --- | --- | --- | --- |
|  | coefficient | t | coefficient | t | coefficient | t |
| ln*gdp* | 0.082 | 1.14 | 0.84 | 1.28 | 0.92 | 1.33 |
| ln*ind* | 0.24^**^ | 2.56 | 1.31^***^ | 3.16 | 1.54^***^ | 3.56 |
| ln*pop* | -0.04 | -0.98 | 0.94^*^ | 1.68 | 0.91 | 1.56 |
| ln*urb* | 0.10 | 1.31 | 28.09^**^ | 2.30 | 29.09^**^ | 2.31 |
| ln*urb^2^* | 0.83^*^ | 1.93 | 16.34^**^ | 1.99 | 17.17^**^ | 2.03 |
| ln*are* | 0.03 | 0.52 | 0.02 | -0.05 | 0.06 | 0.12 |
| ln*stu* | -0.10 | -0.65 | -6.11^**^ | -2.33 | -6.22^**^ | -2.31 |
| ln*rd* | 0.04 | 1.08 | -0.13 | -0.30 | -0.08 | -0.19 |

Note:^*^ *p* < 0.1, ^**^ *p* < 0.05, ^***^ *p* < 0.01.

Thanks!

**Reviewer #1comments6:**

Suggest adding policy implications in the Discussion or Conclusion section to highlight the practical significance of this study, and echo the problem the author aims to address - "to provide a reference for the government to formulate and improve atmospheric environmental protection policies".

**Response:** Thanks for your valuable comments and suggestions. We have added policy recommendations in the conclusion section. The details are as follows:

**Conclusion** **and policy suggestion**

After analyzing the distribution and spatial and temporal characteristics of ozone pollution in China, this paper explores the driving mechanism of ozone pollution in the context of urbanization. Using the spatial econometric model and quantitative method, this paper analyzes the regional differences, temporal and spatial distribution patterns, influencing factors, and spatial spillover effects of ozone pollution in China, with the conclusions as follows:

First, the lowest value of ozone concentration in China's ambient air from 2015 to 2019 was in 2015, and the average annual increase was 1.68*㎍/m^3^*. There is a significant spatial autocorrelation in the level of ozone pollution. Among them, Beijing, Shanghai, Tianjin, Shandong, Henan, Hebei, Inner Mongolia, Anhui, Liaoning and Jiangsu are always the high value areas of ozone concentration, and the spatial distribution of the agglomeration area in the eastern expansion of the "east-heavy and west-light" and "south-short and north-high" characteristics, which indicates that ozone pollution has obvious regional and agglomeration characteristics.

Second, selecting a spatial econometric model to quantitatively identify the key factors influencing ozone concentration in ambient air, we found that *stu* and *rd* have a negative effect on the increase of ozone concentration, while *ind*, *urb*, *gdp* and *are* have a positive effect on the increase of ozone concentration. Studies have shown that there are not only direct effects that affect the level of local ozone pollution, but also indirect effects that affect the level of adjacent ozone pollution also known as spillover effects.

Last, there is also spatial heterogeneity in the factors influencing the increase in ozone concentration. The regression model analysis shows that the inhibitory effect of *stu* and *rd* on the increase of ozone concentration has been reduced, and the promotion effect of *urb* rate, *are* and *gdp* on the increase of ozone concentration has accumulated. The importance influencing factors of ozone concentration from large to small are *stu*, *ind*, *urb*, *gdp*, *are*, *pop* and *rd*.

Based on the research conclusions of this paper, to further reduce ozone pollution, it is necessary to reduce the negative effects brought about by the development of urbanization in China, so as to promote the high-quality development of urban economy. Therefore, we propose the following countermeasures and suggestions:

First, it is necessary to strengthen and improve the system design of ozone pollution control. Ozone pollution sources are mainly from nitrogen oxides and VOC precursors. It should include nitrogen oxides and VOC precursors in the national emission reduction index system to control their emissions. Since the precursors responsible for ozone pollution also contribute to PM_2.5_ emissions, combining ozone pollution control and PM_2.5_ control can enhance interactive data monitoring between the two and facilitate data sharing. Ozone pollution is optical pollution, and the amount of pollution is large when the temperature is high. Therefore, in the process of controlling climate warming, it is also necessary to consider the influence and change of ozone concentration and strengthen the synchronization of climate change control and ozone control.

Second, establish a regional ozone pollution collaborative governance mechanism. Ozone pollution has significant spatial dependence characteristics. The development strategy of environmentally friendly urbanization formulated separately in any region has a limited inhibitory effect on ozone pollution. Only by strengthening inter-regional coordination and cooperation and implementing joint prevention and control can ozone be treated more effectively. The design and improvement of inter-regional ecological protection compensation mechanisms should be strengthened, and financial transfers to areas with serious ozone pollution should be increased.

Third, develop differentiated industrial structure adjustment strategies. There are huge differences in regional economic development level, factor endowment, industrial structure, and urbanization degree, and ozone pollution shows obvious spatial heterogeneity. Therefore, we should adopt differentiated industrial restructuring strategies to achieve the reduction of ozone pollution. For areas with high urbanization levels, we should accelerate the elimination of high pollution and high energy consumption industries, promote the transformation of resource-intensive industries and labor-intensive industries to technology-intensive industries, vigorously develop high-tech industries and modern service industries such as finance and logistics, and accelerate the proportion of the tertiary industry in the national economy.

Last, strengthen the research development and promotion of environmental protection technology. Technological progress is an important means to improve the efficiency of resource utilization and improve the ecological environment. In the process of urbanization, it is necessary to accelerate the research and development, application and promotion of environmental protection technology, and realize the coordinated development of urbanization and ozone governance.

Thanks!

**Reviewer #1comments7:**

There are issues regarding formatting conventions (e.g. characters in formulas should be italic, superscripts for units, the "3" in O3 should be a subscript, etc.), table/figure formatting (e.g. font size in figures is too small, affecting readability), and other details that could be standardized. I will not list them one by one here, but the author should thoroughly proofread and edit the manuscript.

**Response:** Thanks for your valuable comments and suggestions. We have added policy recommendations in the conclusion section. Thank you very much for your suggestions on the details that exist, we have proofread and revised the entire text. The specific details are reflected in the revised draft. Thanks once more!

**Reviewer #2comments1:**

In the "Introduction" section, the introduction expounds too much very general and well-known information, and the importance and significance of the research are not highlighted.

**Response:** Thanks for your valuable comments and suggestions. We have added the importance and significance of the study in the first paragraph of the introduction. The details are as follows:

In the early days, China's economic development was driven at the expense of serious environmental pollution. With the intensification of atmospheric environmental pollution, strengthening environmental governance has become a rigid demand for China's economic development. In 2013, the Chinese government launched the "Air Pollution Prevention and Control Action Plan" with PM_2.5_ as the main control index, among which PM_10_, SO_2_, NO_2_, PM_2.5_, and CO pollutant emissions have been effectively improved. The increase in ozone concentration in ambient air has become more and more serious in recent years [1-3]. Ozone plays a decisive role in the atmospheric environment, and high-altitude ozone is the Earth's protective film against ultraviolet burns on human skin. The environmental effects of surface ozone on humans are mainly due to the fact that ozone is very harmful to biological life and health [4], and high ozone concentrations lead to increased mortality from cardiovascular and respiratory diseases in the population [5-6]. From the perspective of changes in urban ozone concentrations, the formation of ozone in the urban environment is complex. Ozone pollution caused by human activities is mainly caused by photochemical reactions of pollutants such as nitrogen oxides produced by production, construction, transportation, and fuel combustion [7]. At this stage, ozone pollution control is facing severe challenges. Since the "Twelfth Five-Year Plan" period, China has given great importance to the prevention and control of air pollution and has considered improving air quality as a major livelihood project. The concentration of a number of air pollutants focusing on PM_2.5_ has dropped significantly, and the number of days of heavy air pollution has also decreased significantly [8]. However, near-surface Ozone pollution has become increasingly prominent [9], especially in summer has become the primary factor leading to excessive air quality in some cities [10]. Therefore, how to control the concentration of PM2.5 and at the same time curb the increase of Ozone is the current air pollution prevention, and control of the urgent need to solve the problem, should attract the attention of the government and the public.

Thanks!

**Reviewer #2comments2:**

In the "Introduction" section, The author lacks references when expounding the research background.

**Response:** Thanks for your valuable comments and suggestions. We have improved the introduction by adding a reference in the second paragraph. The details are as follows:

Urbanization is the process of economic, social, land, and population transformation from countryside to towns in the course of a country's development. Among them, the core of urbanization is population urbanization, and the carrier of urbanization is land urbanization [11]. With the intensification of atmospheric environmental pollution, scholars from all walks of life have studied the effect of pollution reduction and carbon reduction from multiple perspectives and found that urbanization is an important factor affecting the atmospheric environment [12]. "China's New Urbanization Report 2012" points out that the non-intensive and crude production of China's urbanization focuses on the quantity and scale of urban development, which ignores the value of environmental resources and is an important cause of the decline in the quality of the atmospheric environment. The problem of air pollution in China has changed from single pollutant pollution to compound pollution, which is increasing with the rapid development of urbanization in China [13] [14]. Civerolo et al. [15] used the SLEUTH model to extrapolate New York City's land cover data from 1990 to the next 2050 and predicted that future urbanization in New York will lead to elevated ozone concentrations. Scholars agree with the conclusion that urbanization has an impact on air pollution. Carbon emissions and haze are the main control gases. Scholars from all walks of life have done a lot of research on them, and the methods are diverse. From the existing literature, the STIRPAT model [16], Dubin model [17], DPSIR model [18], the geographically weighted regression model (GWR) [19], geographical detector [20], and so on are mainly used to study the development law of air pollution. Chameides [21] believed that the urban heat island effect caused by urbanization is the main reason for the increase in urban ozone concentration. Many scholars use the WRF Chem model to check ozone pollution, and it shows that urbanization increases ozone concentration. [22-23]. Overall, the existing research results are relatively rich and provide an empirical basis for exploring the relationship between urbanization and air pollution. However, the exploration of urbanization on ozone pollution is still relatively small. In recent years, the causes of atmospheric pollution have strengthened from a purely environmental problem to a complex ecosystem problem of population, land, economy, and society. Current urbanization studies mostly use land cover data to select indicators and fail to consider other factors that influence ozone concentration. There is also limited research on the atmospheric environment effect from the perspective of urbanization. Therefore, understanding the characteristics of spatial and temporal changes in ozone and the driving mechanisms from the perspective of urbanization is essential to further promote the harmonious development of nature and society.

Thanks!

**Reviewer #2comments3:**

For readers to quickly catch your contribution, it would be better to highlight major difficulties and challenges, and your original achievements to overcome them, in a clearer way in abstract and introduction.

**Response:** Thanks for your valuable comments and suggestions. We improved the introduction by adding the main difficulties and challenges of the research. The details are as follows:

Considering all the above explanations, the present study proposes to address the following questions: i) The distribution characteristics of ozone in time and space. ii) Under the background of urbanization development, the driving mechanism of ozone pollution is analyzed (from the four urbanization development directions of economy, population, land, and society). iii) To unify the distribution of factors affecting ozone concentration by urbanization development in all provinces of the country. In addition, the distribution and driving mechanisms of ozone pollution from the perspective of China's urbanization are less frequently studied. To answer these questions above, we attempt to use ozone concentration data and statistical yearbook data of 31 provinces in China to study the driving mechanism of ozone pollution under the context of urbanization. We intensify on the impact of urbanization indicators on ozone and analyze the geographical spatial distribution of urbanization impact factors on the ozone impact index. By citing urbanization, hope to be more comprehensive on the causes of China's distribution characteristics of ozone pollution and how to solve this problem are discussed, and thus draw more accurate results and set forward more constructive suggestions. The intensification of atmospheric environmental pollution in China has attracted extensive attention from the government and academia, but the domestic existing literature, focuses on exploring the micro-channels of urbanization on ozone pollution, and the index selection is based on urban land cover data. The research on the impact of other influencing factors of urbanization on ozone concentration is indeed more insufficient. Therefore, this study aims to empirically analyze the impact mechanism and driving factors of ozone pollution in the context of urbanization in China using the dynamic spatial Durbin model and the spatio-temporal geographically weighted regression model. Producing a new explanation for the causes of the spatial and temporal distribution characteristics of ozone pollution, producing a basis for regional ozone and pollution prevention and control measures, has important theoretical and practical relevance for the government to formulate and improve atmospheric environmental protection policies, and how the government will carry out macro in the future.

Thanks!

**Reviewer #2comments4:**

More explanation is needed for where there is a research gap and what the goals of the research are. The research gap and the goals of the research are not explained in detail which leads to the reader missing the significance of the research.

**Response:** Thanks for your valuable comments and suggestions. We have improved the introduction by adding the goals, gaps and innovations of the research in the last paragraph. The details are as follows:

Considering all the above explanations, the present study proposes to address the following questions: i) The distribution characteristics of ozone in time and space. ii) Under the background of urbanization development, the driving mechanism of ozone pollution is analyzed (from the four urbanization development directions of economy, population, land, and society). iii) To unify the distribution of factors affecting ozone concentration by urbanization development in all provinces of the country. In addition, the distribution and driving mechanisms of ozone pollution from the perspective of China's urbanization are less frequently studied. To answer these questions above, we attempt to use ozone concentration data and statistical yearbook data of 31 provinces in China to study the driving mechanism of ozone pollution under the context of urbanization. We intensify on the impact of urbanization indicators on ozone and analyze the geographical spatial distribution of urbanization impact factors on the ozone impact index. By citing urbanization, hope to be more comprehensive on the causes of China's distribution characteristics of ozone pollution and how to solve this problem are discussed, and thus draw more accurate results and set forward more constructive suggestions. The intensification of atmospheric environmental pollution in China has attracted extensive attention from the government and academia, but the domestic existing literature, focuses on exploring the micro-channels of urbanization on ozone pollution, and the index selection is based on urban land cover data. The research on the impact of other influencing factors of urbanization on ozone concentration is indeed more insufficient. Therefore, this study aims to empirically analyze the impact mechanism and driving factors of ozone pollution in the context of urbanization in China using the dynamic spatial Durbin model and the spatio-temporal geographically weighted regression model. Producing a new explanation for the causes of the spatial and temporal distribution characteristics of ozone pollution, producing a basis for regional ozone and pollution prevention and control measures, has important theoretical and practical relevance for the government to formulate and improve atmospheric environmental protection policies, and how the government will carry out macro in the future.

Thanks!

**Reviewer #2comments5:**

Inadequate methodology rationale: Justification for the model design and method selection was insufficient and merits elaboration.

**Response:** Thanks for your valuable comments and suggestions. We have elaborated on the methodology section, adding the methodological basis and rationale. The details are as follows:

**Ozone response effect model of urbanization process.** The IPAT model is mainly used to reflect the impact of human activities on atmospheric environmental pressure and to analyze the impact of ozone concentration (*I*), population size (*P*), affluence (*A*), and technological progress (*T*). We established STIRPAT model in the IPAT model, which is mainly used to study various environmental impact indicators at home and abroad. To study the relationship between the urbanization process and ozone concentration in ambient air in China, the traditional data model is improved [26-28] to test the relationship, direction, and intensity of mutual influence of spatial variables. Based on the STIRPAT model, a spatial econometric model of the relationship between the urbanization process and ozone concentration in ambient air is constructed [28]. The specific model expressions are as follows.

**Response Results**

In order to determine the specific form of the model, this paper carried out the LM test, LR test, and Hausman test. According to the LM results in Table 3, LM rejects the null hypothesis, and the results tend to be the spatial error model (SEM). Further, the LR test is used to verify the model, and it is found that the null hypothesis is rejected at the 1% level. The Hausman test showed that the null hypothesis was rejected at the 1% level. Therefore, the fixed effect spatial error model (SEM) was finally selected for the experiment.

**Table 3. LM test of spatial panel model**

| LM test | | | |
| --- | --- | --- | --- |
|  | Statistic | df | *p*-value |
| Spatial error |  |  |  |
| Moran’s *I* | 113.175 | 1 | 0.000 |
| Lagrange multiplier | 8.510 | 1 | 0.004 |
| Robuet Lagrange multiplier | 8.680 | 1 | 0.003 |
| Spatial lag |  |  |  |
| Lagrange multiplier | 0.826 | 1 | 0.364 |
| Robuet Lagrange multiplier | 0.995 | 1 | 0.319 |

Thanks!

**Reviewer #2comments6:**

This article has obtained some interesting findings through the models, but these findings need to be further verified from theory or actual conditions. Also, further highlight the contribution of this article.

**Response:** Thanks for your valuable comments and suggestions. We have added references in the empirical analysis section to validate the findings of this paper. The details are as follows:

The presence or absence of spatial autocorrelation features between variables determines whether the STARPAT model of equation (4) can incorporate spatial effects. In order to accurately analyze the impact of urbanization on ozone pollution and test whether there is spatial autocorrelation between urbanization rate and ozone pollution, we calculated the Moran’*I* values for both as shown in Table 2. We can observe from the table that environmental pollution levels in 2015-2019 mostly have positive Moran’*I* values. This indicates that there is a significant positive spatial autocorrelation in the level of ozone pollution among 31 provinces in China. In addition, the Moran’*I* value of ozone in 2015 was 0.027, which rose to 0.244 in 2019, indicating that the spatial agglomeration effect of ozone pollution in China was becoming more and more obvious.

**Table 2. 2015-2019 National Ozone Pollution Level Moran'*I*** Index and its inspection results

|  | 2015 | | 2016 | | 2017 | | 2018 | |  | 2019 |
| --- | --- | --- | --- | --- | --- | --- | --- | --- | --- | --- |
| Moran*^’^I* | I | P | I | P | I | P | I | P | I | P |
| ln*O_3__8h* | 0.027 | 0.220 | 0.128 | 0.019 | 0.209 | 0.001 | 0.166 | 0.005 | 0.244 | 0.000 |
| ln*gdp* | 0.037 | 0.180 | 0.028 | 0.213 | 0.025 | 0.222 | 0.018 | 0.251 | 0.014 | 0.269 |
| ln*ind* | -0.039 | 0.465 | -0.042 | 0.451 | 0.063 | 0.339 | -0.050 | 0.403 | -0.057 | 0.366 |
| ln*pop* | -0.081 | 0.269 | -0.128 | 0.112 | 0.146 | 0.073 | -0.153 | 0.060 | -0.121 | 0.128 |
| ln*urb* | 0.026 | 0.212 | 0.027 | 0.209 | 0.030 | 0.198 | 0.030 | 0.192 | 0.035 | 0.175 |
| ln*urb^2^* | 0.006 | 0.271 | 0.007 | 0.267 | 0.010 | 0.253 | 0.011 | 0.240 | 0.015 | 0.211 |
| ln*are* | 0.080 | 0.066 | 0.076 | 0.074 | 0.064 | 0.097 | 0.061 | 0.104 | 0.058 | 0.113 |
| ln*stu* | 0.335 | 0.000 | 0.355 | 0.000 | 0.366 | 0.000 | 0.374 | 0.000 | 0.374 | 0.000 |
| ln*rd* | 0.222 | 0.218 | 0.029 | 0.196 | 0.036 | 0.169 | 0.034 | 0.182 | 0.030 | 0.194 |

The results of Figure 3 further show that the distribution characteristics of near-surface ozone in China are "east-heavy, west-light," "short in the south and high in the north". The spatial distribution trend of diffusion to central China and coastal cities is more prominent in the Beijing, Tianjin, Hebei, and Shandong provinces. Significant spatial heterogeneity characterizes the inter-province. Hebei Province, Tianjin City, and Shandong Province are the key areas of the petrochemical and organic chemical industry in China, and the stock of coal-fired boilers is relatively large. The region has dense traffic, developed logistics, and huge emissions of diesel trucks and fuel vehicles. These factors have expanded VOCs and nitrogen oxide emissions in these areas, forming a medium and long-term pressure on ozone prevention and control in the region. In 2015, the high-value areas of ozone concentration in China were distributed across the eastern region, mainly in Beijing, Shanghai, Shandong, Jiangsu, Qinghai, Zhejiang, Gansu, and Inner Mongolia. In Shanghai, the average annual concentration reached 105*μg/m^3^*. By 2019, the concentration of ozone in China increased compared with 2015, and ozone pollution expanded rapidly in the east, mainly concentrated in North China and East China. Among them, Hebei Province, Tianjin City, and Shanxi Province in North China are the high-value areas of ozone pollution concentration growth in China. The near-surface ozone concentration in Tianjin increased from 77 *μg/m^3^* in 2015 to 106 *μg/m^3^* in 2019. This is because more developed industries, dense populations, and higher emissions of ozone precursors from motor vehicles and industrial sources typically characterize these areas [5].

**Fig 3. The spatial distribution of ozone concentration in China's provinces in 2015 and 2019**

**Response of ozone pollution to urbanization**

**Response Results**

In order to determine the specific form of the model, this paper carried out the LM test, LR test, and Hausman test. According to the LM results in Table 3, LM rejects the null hypothesis, and the results tend to be the spatial error model (SEM). Further, the LR test is used to verify the model, and it is found that the null hypothesis is rejected at the 1% level. The Hausman test showed that the null hypothesis was rejected at the 1% level. Therefore, the fixed effect spatial error model (SEM) was finally selected for the experiment.

**Table 3. LM test of spatial panel model**

| LM test | | | |
| --- | --- | --- | --- |
|  | Statistic | df | p-value |
| Spatial error |  |  |  |
| Moran’s *I* | 113.175 | 1 | 0.000 |
| Lagrange multiplier | 8.510 | 1 | 0.004 |
| Robuet Lagrange multiplier | 8.680 | 1 | 0.003 |
| Spatial lag |  |  |  |
| Lagrange multiplier | 0.826 | 1 | 0.364 |
| Robuet Lagrange multiplier | 0.995 | 1 | 0.319 |

**Table 4.** Spatial error model under nested matrix

| Variable | Direct effect | | Indirect effect | | Gross effect | |
| --- | --- | --- | --- | --- | --- | --- |
|  | coefficient | t | coefficient | t | coefficient | t |
| ln*gdp* | 0.082 | 1.14 | 0.84 | 1.28 | 0.92 | 1.33 |
| ln*ind* | 0.24^**^ | 2.56 | 1.31^***^ | 3.16 | 1.54^***^ | 3.56 |
| ln*pop* | -0.04 | -0.98 | 0.94^*^ | 1.68 | 0.91 | 1.56 |
| ln*urb* | 0.10 | 1.31 | 28.09^**^ | 2.30 | 29.09^**^ | 2.31 |
| ln*urb^2^* | 0.83^*^ | 1.93 | 16.34^**^ | 1.99 | 17.17^**^ | 2.03 |
| ln*are* | 0.03 | 0.52 | 0.02 | -0.05 | 0.06 | 0.12 |
| ln*stu* | -0.10 | -0.65 | -6.11^**^ | -2.33 | -6.22^**^ | -2.31 |
| ln*rd* | 0.04 | 1.08 | -0.13 | -0.30 | -0.08 | -0.19 |

Note:^*^ *p* < 0.1, ^**^ *p* < 0.05, ^***^ *p* < 0.01.

Based on the SEM partial differential method, the spillover effect model of urbanization on ozone pollution is constructed (Table 4). The total effect can be decomposed into two parts. One is the direct effect, which indicates the impact of local urbanization development on ozone pollution in the region. The other is the indirect effect, which indicates the impact of local urbanization development on ozone pollution in neighboring areas. Overall the direction of the coefficients of the indicators of the total effect is consistent with the indirect effect. The results of the analysis of the specific individual indicators are:

i) The first-order coefficient of the total effect of urbanization rate (*urb*) is 29.09, and the second-order coefficient of urbanization rate is 17.17. All have significant spillover effects at the 5% level. Production, transportation, fuel combustion and other activities caused by the scale effect of the urban population are the main causes of ozone pollution in the region [12].

ii) The coefficient of economic growth (*gdp*) is positive, with a coefficient of 0.08 for the direct effect and 0.84 for the indirect effect, indicating that economic development contributes more to ozone pollution in neighboring areas than locally. This may be due to the pressure of regional emission reduction, which brings pollution problems to neighboring areas in the form of pollution industry transfer, resulting in higher ozone pollution concentration in neighboring areas than in local areas.

iii) Industrial structure (*ind*) is positively correlated with ozone concentration and is significant at 1 per cent level. It shows that for every 1% increase in industrial structure the ozone concentration in the area increases by 1.54*㎍/m^3^*. China's coast is a key agglomeration of heavy industrial industries. In the process of industrial development of the ecological environment is poor, the industrial structure of the heavy or transport structure and other factors are important reasons for the increase in regional ozone concentrations [30]. Emission reduction policies tailored to different regional conditions are important for the ozone environment [31].

iv) The direct effect coefficient of population density (*pop*) is -0.04, the indirect effect coefficient is 0.94, and the ozone pollution in the adjacent areas is significant at the 10% level. It shows that the agglomeration of population in the region can reduce the local ozone pollution, but it can promote the ozone pollution in the adjacent area. The reason is that population agglomeration can promote the improvement of technological level and reduce ozone concentration to a certain extent, but the activities of motor vehicle exhaust and industrial emissions caused by the scale effect of population will lead to the increase of ozone concentration in ambient air. In addition, the influx of population to economically developed areas and the neighboring underdeveloped areas due to population loss leads to lower emission reduction technology than developed areas, which may also lead to increased ozone pollution.

v) Some scholars believe that urban land expansion aggravates ozone pollution. The coefficients of land expansion (*are*) in this study are positive, which is consistent with most research conclusions [17, 21-22]. It shows that the expansion of an urban scale brings huge energy consumption and has a positive impact on the increase of ozone concentration. Factors such as unreasonable urban construction and management measures can lead to a decline in urban environmental quality.

vi) The coefficients on educational attainment (*stu*) were all negative, and the total effect coefficient was -6.22, significant at the 5 per cent level, indicating that a 5 percent point increase in the proportion of university students was associated with a reduction in ambient air ozone concentrations of 6.22*㎍/m^3^*. This shows that the ratio of college students represents the level of social urbanization in China, which affects social consciousness to a certain extent [17] and inhibits ozone pollution.

vii) The effect of R&D intensity (*rd*) on local ozone concentrations is positive and the effect on neighboring areas is negative. This may be the reason technological advances are based on the actual production process [32]. In terms of technology, technology can be divided into abatement technology and production technology, and the direction of research and development funding greatly affects ozone concentrations.

Thanks!

**Reviewer #2comments7:**

The article lacks an important discussion link, in which the author should focus on describing the differences between the article study and other scholars' studies, thus highlighting the relevance and academic value of the article, the following literature should be helpful for your research：(1) Reduction pathways identification of Agricultural Water Pollution in Hubei Province, China. (2)Coordination of the Industrial-Ecological Economy in the Yangtze River Economic Belt, China.

**Response:** Thanks for your valuable comments and suggestions. We have rewritten the discussion section with reference to these two articles provided in the album, and the additions focus on describing the differences between the article's study and other scholars' studies. The details are as follows:

**Discussion on control variables and research methods**

Li et al. [25] used the data of 31 provinces from 2000 to 2017 as samples to test the impact mechanism of urbanization on environmental pollution. The results show that urbanization has a significant spatial spillover effect on environmental pollution, and the spillover effect between regions is greater than the local effect within the region. Shao et al. [32] tested the heterogeneous impact of the two urbanization promotion modes of compact intensive and scale expansion on haze pollution. Lou et al. [40] conducted an empirical study in Wuyi County. The results showed that there was a significant positive correlation between the urbanization development index (except for total industrial output value) and ozone mass concentration, and urbanization development aggravated ozone pollution. All the above studies illustrate that urbanization due to the economy and land leads to increased ozone pollution. However, this paper argues that it is unrealistic to reflect the impact of urbanization on ozone in terms of economics and land alone. In recent years, the causes of atmospheric pollution have strengthened from a purely environmental problem to a complex ecosystem problem of population, land, economy, and society. The urbanization indicators selected in this study are more diversified, not only validated with urban land cover data but also considering the impact of other influencing factors on ozone pollution concentration. Therefore, it differs from previous studies. In this work, the author not only analyzes the changes of various air indicators over the years from the perspective of time, but also uses spatial econometric models and geographically weighted regression models to analyze the impact of urbanization indicators on ozone, and analyzes the impact of urbanization impact factors on ozone Impact index on the geographical spatial distribution. The results of urbanization Moran's I show that the autocorrelation between ozone and various influencing factors (land, population, economic, and social urbanization) is statistically significant. This makes the study more comprehensive.

In terms of research methodology, in order to discern the specific form of the model, this study conducted LM test, LR test with Hausman test. The LM rejected the original hypothesis, and the results favored the spatial error model (SEM), and the model was further validated by the LR test, which was found to reject the original hypothesis at the 1% level. And the original hypothesis is rejected at 1% level as shown by Hausman test. The fixed-effects spatial error model (SEM) was finally chosen for the experiment. The spatial error model (SEM) is based on selected criteria (log-likelihood, AIC, SBC) and is relatively widely used because it incorporates both endogenous and exogenous interaction effects.

Thanks!

**Reviewer #2comments8:**

Ambiguous conclusions: Conclusions were somewhat vague; more in-depth synthesis to derive more definitive conclusions would be advantageous.

**Response:** Thanks for your valuable comments and suggestions. We have reorganized the conclusions along with recommendations for responses that can better highlight the significance of the study. The details are as follows:

**Conclusion** **and policy suggestion**

After analyzing the distribution and spatial and temporal characteristics of ozone pollution in China, this paper explores the driving mechanism of ozone pollution in the context of urbanization. Using the spatial econometric model and quantitative method, this paper analyzes the regional differences, temporal and spatial distribution patterns, influencing factors, and spatial spillover effects of ozone pollution in China, with the conclusions as follows:

First, the lowest value of ozone concentration in China's ambient air from 2015 to 2019 was in 2015, and the average annual increase was 1.68*㎍/m^3^*. There is a significant spatial autocorrelation in the level of ozone pollution. Among them, Beijing, Shanghai, Tianjin, Shandong, Henan, Hebei, Inner Mongolia, Anhui, Liaoning and Jiangsu are always the high value areas of ozone concentration, and the spatial distribution of the agglomeration area in the eastern expansion of the "east-heavy and west-light" and "south-short and north-high" characteristics, which indicates that ozone pollution has obvious regional and agglomeration characteristics.

Second, selecting a spatial econometric model to quantitatively identify the key factors influencing ozone concentration in ambient air, we found that *stu* and *rd* have a negative effect on the increase of ozone concentration, while *ind*, *urb*, *gdp* and *are* have a positive effect on the increase of ozone concentration. Studies have shown that there are not only direct effects that affect the level of local ozone pollution, but also indirect effects that affect the level of adjacent ozone pollution also known as spillover effects.

Last, there is also spatial heterogeneity in the factors influencing the increase in ozone concentration. The regression model analysis shows that the inhibitory effect of *stu* and *rd* on the increase of ozone concentration has been reduced, and the promotion effect of *urb* rate, *are* and *gdp* on the increase of ozone concentration has accumulated. The importance influencing factors of ozone concentration from large to small are *stu*, *ind*, *urb*, *gdp*, *are*, *pop* and *rd*.

Based on the research conclusions of this paper, to further reduce ozone pollution, it is necessary to reduce the negative effects brought about by the development of urbanization in China, so as to promote the high-quality development of urban economy. Therefore, we propose the following countermeasures and suggestions:

First, it is necessary to strengthen and improve the system design of ozone pollution control. Ozone pollution sources are mainly from nitrogen oxides and VOC precursors. It should include nitrogen oxides and VOC precursors in the national emission reduction index system to control their emissions. Since the precursors responsible for ozone pollution also contribute to PM_2.5_ emissions, combining ozone pollution control and PM_2.5_ control can enhance interactive data monitoring between the two and facilitate data sharing. Ozone pollution is optical pollution, and the amount of pollution is large when the temperature is high. Therefore, in the process of controlling climate warming, it is also necessary to consider the influence and change of ozone concentration and strengthen the synchronization of climate change control and ozone control.

Second, establish a regional ozone pollution collaborative governance mechanism. Ozone pollution has significant spatial dependence characteristics. The development strategy of environmentally friendly urbanization formulated separately in any region has a limited inhibitory effect on ozone pollution. Only by strengthening inter-regional coordination and cooperation and implementing joint prevention and control can ozone be treated more effectively. The design and improvement of inter-regional ecological protection compensation mechanisms should be strengthened, and financial transfers to areas with serious ozone pollution should be increased.

Third, develop differentiated industrial structure adjustment strategies. There are huge differences in regional economic development level, factor endowment, industrial structure, and urbanization degree, and ozone pollution shows obvious spatial heterogeneity. Therefore, we should adopt differentiated industrial restructuring strategies to achieve the reduction of ozone pollution. For areas with high urbanization levels, we should accelerate the elimination of high pollution and high energy consumption industries, promote the transformation of resource-intensive industries and labor-intensive industries to technology-intensive industries, vigorously develop high-tech industries and modern service industries such as finance and logistics, and accelerate the proportion of the tertiary industry in the national economy.

Last, strengthen the research development and promotion of environmental protection technology. Technological progress is an important means to improve the efficiency of resource utilization and improve the ecological environment. In the process of urbanization, it is necessary to accelerate the research and development, application and promotion of environmental protection technology, and realize the coordinated development of urbanization and ozone governance.

Thanks once more!

**Editors #comments1:**

Please ensure that your manuscript meets PLOS ONE's style requirements, including those for file naming. The PLOS ONE style templates can be found at

<https://journals.plos.org/plosone/s/file?id=wjVg/PLOSOne_formatting_sample_main_body.pdf> and

<https://journals.plos.org/plosone/s/file?id=ba62/PLOSOne_formatting_sample_title_authors_affiliations.pdf>

**Response:** Thanks for your valuable comments and suggestions. We have modified the format of the manuscript and ensured that your manuscript meets PLOS ONE's style requirements.

Thanks!

**Editors #comments2:**

Thank you for stating the following financial disclosure: "This work was supported by the National Social Science Foundation of China (grant numbers: 16BJY039), Harbin Normal University Graduate Student Innovation Project (grant numbers: HSDBSCX2023-12)." Please state what role the funders took in the study. If the funders had no role, please state:" The funders had no role in study design, data collection and analysis, decision to publish, or preparation of the manuscript." If this statement is not correct you must amend it as needed. Please include this amended Role of Funder statement in your cover letter; we will change the online submission form on your behalf.

**Response:** Thanks for your valuable comments and suggestions. We have added this information to our cover letter.

Thanks!

**Editor #comments3:**

We note that Figure 4 in your submission contain map/satellite images which may be copyrighted. All PLOS content is published under the Creative Commons Attribution License (CC BY 4.0), which means that the manuscript, images, and Supporting Information files will be freely available online, and any third party is permitted to access, download, copy, distribute, and use these materials in any way, even commercially, with proper attribution. For these reasons, we cannot publish previously copyrighted maps or satellite images created using proprietary data, such as Google software (Google Maps, Street View, and Earth). For more information, see our copyright guidelines:

<http://journals.plos.org/plosone/s/licenses-and-copyright>. We require you to either (a) present written permission from the copyright holder to publish these figures specifically under the CC BY 4.0 license, or (b) remove the figures from your submission:

a. You may seek permission from the original copyright holder of Figure 4 to publish the content specifically under the CC BY 4.0 license. We recommend that you contact the original copyright holder with the Content Permission Form (<http://journals.plos.org/plosone/s/file?id>=7c09

content-permission-form.pdf) and the following text:“I request permission for the open-access journal PLOS ONE to publish XXX under the Creative Commons Attribution License (CCAL) CC BY 4.0 (http://creativecommons.org/licenses/by/4.0/). Please be aware that this license allows unrestricted use and distribution, even commercially, by third parties. Please reply and provide explicit written permission to publish XXX under a CC BY license and complete the attached form.” Please upload the completed Content Permission Form or other proof of granted permissions as an "Other" file with your submission. In the figure caption of the copyrighted figure, please include the following text: “Reprinted from [ref] under a CC BY license, with permission from [name of publisher], original copyright [original copyright year].”

**Response:** Thanks for your valuable comments and suggestions. We have filled out the Content_Copyright_License_Form and completed the signature on it. Also entered the text in Word called Text Note. All are uploaded as "other" files.

Thanks once more!
