# Supplementary material for: Spatialtemporal evolution characteristics of ozone in China and its response to urbanization
Source: PLoS One. 2024 May 31;19(5):e0300185. doi: 10.1371/journal.pone.0300185 (PMC11142485; doi:10.1371/journal.pone.0300185)
Supplement: Attachment — Submitted filename: Response to reviewers .doc [file pone.0300185.s004.doc]

Thank you for your letter and for the reviewers' and editors' comments concerning our manuscript. These comments are very helpful for revising and improving our paper. We have studied comments carefully and have made corrections which we hope meet with approval.

**Reviewer #1comments1:**

The manuscript has been revised in accordance with the comments and is essentially ready for publication. It is hoped that the authors will further revise the formatting of the manuscript (e.g., clarity of figures, formatting of references, etc.) in accordance with the requirements of the journal. References 41-43 are not labeled in the manuscript, please revise it.

**Response:** Thanks for your valuable comments and suggestions. We have re-formatted the manuscript as required by the journal and have also made changes to the references. The specific changes are reflected in the manuscript.

Thank you very much for your valuable advice!

**Reviewer #2comments1:**

The title of the article needs to be revised.

**Response:** Thanks for your valuable comments and suggestions. The present study proposes to address the following questions: i) The distribution characteristics of ozone in time and space. ii) Under the background of urbanization development, the driving mechanism of ozone pollution is analyzed (from the four urbanization development directions of economy, population, land, and society). iii) To unify the distribution of factors affecting ozone concentration by urbanization development in all provinces of the country. To answer these questions above, we attempt to use ozone concentration data and statistical yearbook data of 31 provinces in China to study the driving mechanism of ozone pollution under the context of urbanization. Exploring the response of ozone pollution to urbanization.

In summary, we believe that this title "Spatialtemporal evolution characteristics of ozone in China and its response to urbanization" is consistent with the main thrust of the manuscript's.

Thanks!

**Reviewer #2comments2:**

The innovation of this paper needs to be highlighted in the abstract, and the background of the paper needs to modify.

**Response:** Thanks for your valuable comments and suggestions. We have reworked and rewritten the abstract to add the significance and innovation of the article. The details are as follows:

Abstract

Based on the background of urbanization in China, we used the dynamic spatial panel Durbin model to study the driving mechanism of ozone pollution empirically. We also analyzed the spatial distribution of ozone driving factors using the GTWR. The results show that: i) The average annual increase of ozone concentration in ambient air in China from 2015 to 2019 was 1.68*μg/m^3^*, and 8.39*μg/m^3^* elevated the year 2019 compared with 2015. ii) The Moran's I value of ozone in ambient air was 0.027 in 2015 and 0.209 in 2019, showing the spatial distribution characteristics of "east heavy and west light" and "south low and north high". iii) Per capita GDP industrial structure, population density, land expansion, and urbanization rate have significant spillover effects on ozone concentration, and the regional spillover effect is greater than the local effect. R&D intensity and education level have a significant negative impact on ozone concentration. iv) There is a decreasing trend in the inhibitory effect of educational attainment and R&D intensity on ozone concentration, and an increasing trend in the promotional effect of population urbanization rate, land expansion, and economic development on ozone concentration. Empirical results suggest a twofold policy meaning: i) to explore the causes behind the distribution of ozone from the new perspective of urbanization, and to further the atmospheric environmental protection system and ii) to eliminate the adverse impacts of ozone pollution on nature and harmonious social development.

Thanks!

**Reviewer #2comments3:**

The literature review is not enough, the contribution made by previous studies has not been clearly expressed, and the author needs to introduce the research methods of previous articles to highlight the innovation of this paper.

**Response:** Thanks for your valuable comments and suggestions. We have reworked and supplemented the literature review section to summarise the relevant research methods of scholars and highlight the innovative nature of this paper. The details are as follows:

Urbanization is the process of economic, social, land, and population transformation from countryside to towns in the course of a country's development. Among them, the core of urbanization is population urbanization, and the carrier of urbanization is land urbanization [11]. With the intensification of atmospheric environmental pollution, scholars from all walks of life have studied the effect of pollution reduction and carbon reduction from multiple perspectives and found that urbanization is an important factor affecting the atmospheric environment [12]. "China's New Urbanization Report 2012" points out that the non-intensive and crude production of China's urbanization focuses on the quantity and scale of urban development, which ignores the value of environmental resources and is an important cause of the decline in the quality of the atmospheric environment. The problem of air pollution in China has changed from single pollutant pollution to compound pollution, which is increasing with the rapid development of urbanization in China [13-14]. Civerolo et al. [15] used the SLEUTH model to extrapolate New York City's land cover data from 1990 to the next 2050 and predicted that future urbanization in New York will lead to elevated ozone concentrations. Scholars agree with the conclusion that urbanization has an impact on air pollution. Carbon emissions and haze are the main control gases. Scholars from all walks of life have done a lot of research on them, and the methods are diverse. From the existing literature, the STIRPAT model [16], Dubin model [17], DPSIR model [18], the geographically weighted regression model (GWR) [19], geographical detector [20], and so on are mainly used to study the development law of air pollution. The Theil coefficient, Theil coefficient, coefficient of variation, and spatial autocorrelation (global Moran's *I* index and local G coefficient) are often used to reveal the regional differences and spatial correlation of air pollution intensity. For example, Sun Yaohua et al. [21] explored the differences in carbon emission intensity between provinces and regions in China-based on the Theil index and found that there were differences in carbon emission intensity between provinces in China and accumulated. Zhao Yuntai et al. [22] divided the entire country into eight economic regions and used the Theil index, global autocorrelation Moran's *I*, and cold and hot spot analysis methods to explore the spatial evolution characteristics of regional carbon emission intensity. It was found that the widening difference between regions caused the widening difference of regional carbon emission intensity in the entire country, while the difference within the region was small. The research on the relationship between urbanization and air pollution mainly forms three conclusions: the positive linear relationship between urbanization and air pollution [23-24], the negative linear relationship between urbanization and air pollution [25], and the nonlinear curve relationship between urbanization and air pollution [26]. Among them, scholars have conducted empirical investigations on the different influencing factors of ozone on air pollution, but there are few studies on the impact of ozone distribution in air pollution from the perspective of urbanization.

Thanks!

**Reviewer #2comments4:**

The article was not written following the correct journal's guidelines to be considered for publication. INTORDCUTION→MRTHOD→RESULTS→DISSCUSION→CONCLUSION.

**Response:** Thanks for your valuable comments and suggestions. We have followed the correct journal guidelines for rewriting. The structure is:

INTORDCUTION→MATERIAL AND METHODS→RESULTS→DISCUSSION→CONCLUSION.

Thanks!

**Reviewer #2comments5:**

The discussion should focus on describing the differences between the article study and other scholars' studies, thus highlighting the relevance and academic value of the article, the following literature should be helpful for your research：(1) A Set Pair Analysis Method for Assessing and Forecasting Water Conflict Risk in Transboundary River Basins, (2)Reduction pathways identification of Agricultural Water Pollution in Hubei Province, China.

**Response:** Thanks for your valuable comments and suggestions. We refer to the methodology of these two papers, the introductory section and the main content of the study changed the introductory section and added to the references of this paper. The details are as follows:

Urbanization is the process of economic, social, land, and population transformation from countryside to towns in the course of a country's development. Among them, the core of urbanization is population urbanization, and the carrier of urbanization is land urbanization [11]. With the intensification of atmospheric environmental pollution, scholars from all walks of life have studied the effect of pollution reduction and carbon reduction from multiple perspectives and found that urbanization is an important factor affecting the atmospheric environment [12]. "China's New Urbanization Report 2012" points out that the non-intensive and crude production of China's urbanization focuses on the quantity and scale of urban development, which ignores the value of environmental resources and is an important cause of the decline in the quality of the atmospheric environment. The problem of air pollution in China has changed from single pollutant pollution to compound pollution, which is increasing with the rapid development of urbanization in China [13-14]. Civerolo et al. [15] used the SLEUTH model to extrapolate New York City's land cover data from 1990 to the next 2050 and predicted that future urbanization in New York will lead to elevated ozone concentrations. Scholars agree with the conclusion that urbanization has an impact on air pollution. Carbon emissions and haze are the main control gases. Scholars from all walks of life have done a lot of research on them, and the methods are diverse. From the existing literature, the STIRPAT model [16], Dubin model [17], DPSIR model [18], the geographically weighted regression model (GWR) [19], geographical detector [20], and so on are mainly used to study the development law of air pollution. The Theil coefficient, Theil coefficient, coefficient of variation, and spatial autocorrelation (global Moran's *I* index and local G coefficient) are often used to reveal the regional differences and spatial correlation of air pollution intensity. For example, Sun Yaohua et al. [21] explored the differences in carbon emission intensity between provinces and regions in China-based on the Theil index and found that there were differences in carbon emission intensity between provinces in China and accumulated. Zhao Yuntai et al. [22] divided the entire country into eight economic regions and used the Theil index, global autocorrelation Moran's *I*, and cold and hot spot analysis methods to explore the spatial evolution characteristics of regional carbon emission intensity. It was found that the widening difference between regions caused the widening difference of regional carbon emission intensity in the entire country, while the difference within the region was small. The research on the relationship between urbanization and air pollution mainly forms three conclusions: the positive linear relationship between urbanization and air pollution [23-24], the negative linear relationship between urbanization and air pollution [25], and the nonlinear curve relationship between urbanization and air pollution [26]. Among them, scholars have conducted empirical investigations on the different influencing factors of ozone on air pollution, but there are few studies on the impact of ozone distribution in air pollution from the perspective of urbanization.

**References：**

1. Wei Zhao, Bo Gao, Qing Lu, Zhiqiang Zhong, Xiaoming Liang, Ming Liu, Shexia Ma, Jiaren Sun, Laiguo Chen, Shaojia Fan. Ozone pollution trend in Pearl River Delta region from 2006 to 2019. *Environmental Science*. 2021; 42(1), 97-105.
2. Zhongling Hu. Ozone pollution in China is increasing year by year. Ecological Economy. 2020; 36(9), 5-8.
3. Zhenhua Zhang, Jing Wang, Yanchao Feng, Wenjia Tian. Effect of green finance reform and innovation pilot zone on ozone pollution. Chinese Mouth Resources and Environment. 2022; 32(12), 52-65.
4. Shutao Chen, Yong Zhang, Zhenhua Hu, Yanshu Shi, Xiaoshuai Shen. Effects of elevated ozone concentration and soil moisture on respiration temperature sensitivity of soil microorganisms in farmland. Environmental Science. 2012; 33(05), 1476-1483.
5. Tianyue Zhang, Nanchi Shen, Xue Zhao, Xinyu Wang, Wenji Zhao. Spatial-temporal variation characteristics of ozone concentration and population exposure risk assessment in Chengdu-Chongqing urban agglomeration from 2015 to 2019. Journal of Environmental Science. 2021; 41(10), 4188-4199.
6. Weipeng Ye, Miaomiao Liu, Jun Bi. A meta-analysis of the relationship between short-term ozone exposure and human mortality in China. Journal of Environmental Science. 2020; 40(7), 2644-2651.
7. Weiwu Wang, Chao Chen. Quantitative analysis of spatial distribution and influencing factors of urban air pollutants in Hangzhou. Geographical Research. 2008; (2), 241-249+481.
8. Ministry of Ecology and Environment of the People's Republic of China Report on air quality improvement in China (2013-2018). Beijing:Ministry of Ecology and Environment. 2019.
9. Hua Jiang, Jian Gao, Hong LI, Wanghui Chu, Fahe Chai. A preliminary study on the theoretical framework of coordinated prevention and control of air pollution in China. Research of Environmental Sciences. 2022; 35(3), 601-610.
10. Xiaozhe Wang, Sha Zhao, Linghui Guo, Hebing Zhang, Jiangbo Gao. Seasonal variation of ozone in "2+26" cities in Beijing-Tianjin-Hebei region and surrounding areas. Research of Environmental Sciences. 2022; 35(8), 1786-1797.
11. Xi Yang, Xinhai Lu. Driving factors of urban land urbanization in China from the perspective of spatial effects. Chinese Mouth Resources and Environment. 2021; 31(1), 156-164.
12. Jianneng Xiao, Guoming Du, Yiqiang Shi, Youyue Wen, Yao Jie. Spatial-temporal characteristics of ambient air pollution in Xiamen and its correlation with meteorological factors. Journal of Environmental Science. 2016; 36(9), 3363-3371.
13. Wenyuan Niu. Report on New Urbanization in China·2012, Science Press. ISBN: 9787030356246. 2012.
14. Lijian Han, Weiqi Zhou, Steward T P, Weifeng Li, Yuguo Qian. Multicontaminant air pollution in Chinese cities. Bulletin of the World Health Organization. 2018; 96(4), 233. <https://doi.org/10.2471/blt.17.195560>
15. Civerolo K, Hogrefe C, Lynn B, Rosenthal J, Ku J Y, Solecki W, Kinney P. Estimating the effects of increased urbanization on surface meteorology and ozone concentrations in the New York City metropolitan region. Atmospheric environment. 2007; 41(9), 1803-1818. h[ttps://doi.org/10.1016/j.atmosenv.2006.10.076](https://doi.org/10.1016/j.atmosenv.2006.10.076)
16. Rui Huang, Zheng Wang, Guanqun Ding, Yangran Gong, Changxin Liu. Analysis and trend prediction of influencing factors of energy consumption carbon emissions in Jiangsu Province based on STIRPAT model. Geographical Research. 2016; 35(4), 781-789.
17. Shaojian Wang, Jingyuan Zeng, Yongyuan Huang, Chenyi Shi, Peiyu Zhan. The effects of urbanization on CO_2_ emissions in the Pearl River Delta: a comprehensive assessment and panel data analysis. Applied Energy. 2018; 228, 1693-1706. https://doi.org/10.1016/j.apenergy.2018.06.155
18. Qinlin Xiao, Chao Tian, Yanjun Wang, Xiuqing Li, Liming Xiao. Measurement and comparison of urban haze control level and efficiency based on DPSIR model: A case study of 31 cities in North China. Journal of Resources and Ecology. 2020; 11(6), 549-561.
19. Bin Zou, Xin Fang, Huihui Feng, Xiang Zhou. Simplicity versus accuracy for estimation of the PM2.5 concentration: A comparison between LUR and GWR methods across time scales. Journal of Spatial Science. 2021; 66, 279-297. https://doi.org/10.1080/14498596.2019.1624203
20. Lili Wang, Xiaojie Liu, Ding Li, Sun, Y. Geographical exploration of spatial heterogeneity and driving factors in the Yangtze River Economic Belt PM(2.5). Environmental Science. 2022; 43(3), 1190-1200.
21. Yaohua Sun, Weizhou Zhong, Dongrui Qing. Analysis on differences of carbon emission intensity of each province in China based on Theil Index. Finance and Trade Research, 2012; 2(3),1-7.
22. Yuntai Zhao, Xianjin Huang, Taiyang Zhong, Jiawen peng. Spatial pattern evolution of carbon emission intensity from energyconsumption in China. Environmental Science, 2011; 32(11), 3145-3152.
23. Al-Mulali U, Ozturk I, Lean H H. The Influence of economic growth, urbanization, trade openness, ﬁnancial development, and renewable energy on pollution in Europe. Natural Hazards, 2015; 79(1), 621-644. https://doi.org/10.1007/s11069-015-1865-9
24. Bolong Liu, Xiaoling Yuan, Zhanjun Zhang. The Impact of Urbanization on Haze Pollution: An Empirical Analysis Based on China's Provincial Dynamic Panel Data. Urban Development Research,2015; (9),23-27,80.
25. Hong Zhao, Yumeng Chen. Research on the relationship between urbanization process and carbon emission reduction in China. China Soft Science,2013; (3),184-192.
26. Keshen Jiang, Xiaohong Liu. China's economic growth, trade openness, tertiary industry and haze in the process of urbanization: An empirical study based on inter-provincial panel data. Finance &Economics,2017; (3),20-25.
27. Chameides L. Natural hydrocarbons, urbanization, and urban ozone. Journal of Geophysical Research Atmospheres. 1990; 95(D9), 13971.
28. Xuemei Wang, Fei Chen, Zhiyong Wu, Meigen Zhang, Mukul Tewari, Alex Guenther, Christine Wiedinmyer. Impacts of weather conditions modified by urban expansion on surface ozone: Comparison between the Pearl River Delta and Yangtze River Delta regions. Advances in Atmospheric Sciences. 2009; 26(5), 962-972. https://doi.org/10.1007/s00376-009-8001-2
29. Tabla-Hernandez J, Hernandez-Ramirez, A G, Martinez-Tavera E, Rodriguez-Espinosa, P F, Mangas-Ramírez E. Impacts on water quality by in situ induced ozone‑oxygen oxidation in a polluted urban reservoir. *Science of the Total Environment*. 2020; 735, 139364. <https://doi.org/10.1016/j.scitotenv.2020.139364>
30. Shuai Shao, Xin Li, Jianhua Cao. Urbanization and smog control in China. Economic Research Journal. 2019; 54(2), 148-165.
31. Cungui Li. Study on spatial spillover and threshold effect of environmental pollution in China's urbanization. Ecological Economy. 2021; 37(3), 197-206.
32. Lesage, J P, Pace, R K. Introduction to spatial econometrices. Boca Raton, FL.CRC Press. 2009.
33. Yaobin Liu, Huaxi Yuan, Yidai Feng. Spatial spillover and threshold characteristics of industrial agglomeration emission reduction effect. Journal of Mathematical Statistics and Management. 2018; 37(2), 224-234.
34. York R, Rosa E A, Dietz T. STIRPAT, IPAT and IMPACT: Analytic tools for unpacking the driving forces of environmental impacts. Ecological Economics. 2003; 46(3), 351-365. https://doi.org/10.1016/s0921-8009(03)00188-5
35. Shaojian Wang, Yongyuan Huang. Spatial spillover effects and driving factors of carbon emission intensity in Chinese cities. Acta Geographica Sinica. 2019; 74(6), 1131-1148.
36. Yuan Zhao, Lisha Hao, Zuying Yang. Spatial differentiation characteristics and causes of energy efficiency in Jiangsu Province. Acta Geographica Sinica. 2010; 65 (08), 919-928.
37. Xueqin Lin, Dai Wang. Spatial-temporal evolution characteristics and socio-economic driving forces of urban air quality in China. Journal of Geographical Sciences. 2016; 26(11), 1533-1549.
38. Shuai Shao, Xin Li, Jianhua Cao, Lili Yang. Economic Policy Options for Haze Control in China: Based on the Perspective of Spatial Spillover Effects. Economic Research. 2016; 51 (09), 73-88.
39. Bo Yu, Xv Yang, Xiangli Wu, Yuanhe Cao, Ying Cai. Xuewei Wang, Cheng Zhao. Spatial evolution characteristics and influencing factors of urban environmental pollution supervision level in China. Geographical Research. 2019; 38(7), 1777-1790.
40. Hui Li, Xv Yang, Xiangli Wu, Yanhong Jia. The carbon emission effect of the evolution of energy-intensive industrial structure from the perspective of time and space. Journal of Environmental Science. 2021; 41 (5), 2018-2028.
41. Ying Zheng, Fei Lu, Jingru Liu, Xiaoke Wang. A comparative study on the consumption CO2 emissions of fossil energy in typical cities in China and their influencing factors. Acta Ecological Sinica. 2020; 40(10), 3315-3327.
42. Yonglong Lv, Chenchen Wang, Xianghui Cao. Ecological risks of urbanization and their management. Acta Ecological Sinica. 2018; 38(2), 359-370.
43. Xiaogang Huang, Jingbo Zhao, Junji Cao, Song, Y. Spatial-temporal variation characteristics and driving factors of urban O_3_ concentration in China. Environmental Science. 2019; 40(3), 1120-1131.
44. Su Wangxin, Sun Ranhao. Driving factors of carbon emissions of urbanization in typical urban agglomerations in China. Acta Ecological Sinica. 2018; 38(6), 1975-1983.
45. Cardelino, C A, Chameides W L. Natural hydrocarbons, urbanization, and urban ozone. John Wiley & Sons, Ltd. 1990; 95(9), 13971-13979. https://doi.org/10.1029/jd095id09p13971
46. Junwei Lou, Yeping He, Xinheng Du, Li Pan, Haifeng Zhu. Analysis of ozone concentration change characteristics and urbanization impact in Wuyi County. Proceedings of the 2023 Science and Technology Annual Conference of the Chinese Society for Environmental Sciences. Chinese Society for Environmental Sciences, Chinese Society for Environmental Sciences. 2023; 4.
47. Sun Pingjun, Luo Ning. Comparative Analysis of the Quality of Urbanization Structure in Central Cities in Southwest Economic Core Area: A Case Study of Chengdu and Chongqing. SCIENTIA GEOGRAPHICA SINICA. 2021; 41 (06), 1019-1029.
48. Naigang Cao, Ning Luo, Yali Peng, Ju Liu, Keqiu Zhang, Pingjun Sun. Evaluation of Urbanization Development Dynamics Based on Quadrilateral Diamond Structure Model: Evidence from Chongqing, China, in the New Era. Journal of Urban Planning and Development. 2023; 149(3).
49. Chuwei Liu, Xinbo Lian, Jianping Huang. Advances in the study of spatial and temporal distribution and causes of ozone pollution in China. Journal of Arid Meteorology. 2020; 38(03), 355-361.
50. Liang Yuan, Chenyuan Liu, Xia Wu, Weijun He, KongYang, Degefu, Dagmawi M, Ramsey, Thomas S. A Set Pair Analysis Method for Assessing and Forecasting Water Conflict Risk in Transboundary River Basins. Water Resources Management.2023. https://doi.org/10.1007/s11269-023-03698-4
51. Weijun He, Ke Zhang, Kong Yang, Degefu, Dagmawi M, Ramsey, Thomas S. Reduction pathways identification of agricultural water pollution in Hubei Province, China. Ecological Indicators, 2023;153,110-464.

https://doi.org/10.1016/j.ecolind.2023.110464

Thanks!

**Reviewer #2comments6:**

The format of the article should be standardized, and the quality of the figures and tables needs to be improved.

**Response:** Thanks for your valuable comments and suggestions. We have realigned the figures and tables to improve the resolution of Figures 3 and 4. The tables have been changed in accordance with journal requirements. The specific changes are reflected in the manuscript.

Thank you very much for your valuable advice!

**Editors #comments:**

Please review your reference list to ensure that it is complete and correct. If you have cited papers that have been retracted, please include the rationale for doing so in the manuscript text, or remove these references and replace them with relevant current references. Any changes to the reference list should be mentioned in the rebuttal letter that accompanies your revised manuscript. If you need to cite a retracted article, indicate the article’s retracted status in the References list and also include a citation and full reference for the retraction notice.

**Response:** Thanks for your valuable comments and suggestions. We have revised the references to ensure compliance with journal formats.

Thank you very much for your valuable advice!
